# Draft Genome Sequence of *Campylobacter fetus* MMM01, Isolated from a Chronic Kidney Disease Patient with Sepsis

**DOI:** 10.1128/genomeA.01055-15

**Published:** 2015-10-08

**Authors:** Anusha Rohit, Ballamoole Krishna Kumar, Vijay Kumar Deekshit, Praveen Rai, Ramanathan Vijay Kumar, Jayapalan Jayaprakash, Bhat Madhushankara, Iddya Karunasagar, Indrani Karunasagar

**Affiliations:** a Department of Microbiology, The Madras Medical Mission, Chennai, Tamil Nadu, India; b Nitte University Centre for Science Education and Research, Nitte University, Deralakatte, Mangalore, Karnataka, India; c Department of Nephrology, Billroth Hospitals, RA Puram, Chennai, Tamil Nadu, India; d School of Information Science (SOIS), Manipal University, Manipal, India; e FAO Consultant, Subba-Meena, Mangalore, India

## Abstract

*Campylobacter fetus* is a Gram-negative bacterium that has caused several cases of human and animal disease. Here, we report the draft genome sequence of *C. fetus* MMM01, isolated from the blood of a 60-year-old patient with type II diabetes and chronic kidney disease. The sequence has a total length of 1,740,393 bp and an average G+C content of 33.1%. The availability of the draft genome sequence of *C. fetus* MMM01 isolated from a case of chronic kidney disease will contribute to a better understanding of the pathophysiological mechanisms of this organism.

## GENOME ANNOUNCEMENT

*Campylobacter fetus* is a Gram-negative bacterium composed of two subspecies: *C. fetus* subsp. *fetus* and *C. fetus* subsp. *venerealis*, which cause several kinds of diseases in humans and animals (1, 2). The ecological niches of these subspecies are different, and they can be isolated from a variety of different hosts (3, 4). Cases of bacteremia in humans, particularly immunocompromised individuals, due to *C. fetus* have been reported in recent years in several parts of the world (2). Here, we report the draft genome sequence of *C. fetus* MMM01 isolated from the blood of a 60-year-old patient with type II diabetes and chronic kidney disease. The organism was identified as *C. fetus* with a matrix-assisted laser desorption ionization–time of flight mass spectrometry (MALDI TOF MS) system (Bruker Daltonics, Bremen, Germany).

Genomic DNA was extracted from *C. fetus* MMM01 using the QIAamp DNA minikit (Qiagen, Germany). A concentration of 50 ng/µl was used for the genome sequencing, which was performed on an Ion Torrent PGM platform, according to the manufacturer’s protocol (Bioserve Biotechnologies, India). The sequence data were assembled using CLC Genomics Workbench version 6. Structural gene prediction and functional annotation were performed using the Rapid Annotations using Subsystems Technology (RAST) server (5).

A total of 209,580 reads with a mean read length of 150 bp for 200-bp fragmentation chemistry obtained from the Ion PGM were assembled into 143 contigs. The draft genome of *C. fetus* MMM01 has a total length of 1,740,393 bp, with an average G+C content of 33.1%. Annotation of the *C. fetus* MMM01 draft genome was done using RAST and contains 2,278 open reading frames. All 143 contigs from *C. fetus* MMM01 were assembled to *C. fetus* subsp. *fetus* 82-40 (6) using Geneious 6.1.6 and showed a high degree of similarity to *C. fetus* subsp. *fetus* 82-40 (isolated from the blood of a human patient who had a renal transplant). The analysis obtained from RAST revealed 296 subsystems. The annotated genome has 87 genes responsible for virulence, including genes involved in adhesion, toxins, superantigens, bacteriocins, ribosomally synthesized antibacterial peptides, resistance to antibiotics and toxic compounds, invasion, and intracellular survival. The draft genome of *C. fetus* MMM01 has a continuous sequence of approximately 6,445 bp and contains genes coding for the multidrug efflux system (*cmeABC*), including its transcriptional repressor sequences. The genome also contains genes coding for macrolide specific efflux pumps, *macA* and *macB*.

The availability of the draft genome sequence of *C. fetus* MMM01 isolated from a case chronic kidney disease will contribute to a better understanding of the pathophysiological mechanisms of this organism. However, further studies are essential to investigate the virulence potential of this pathogen.

### Nucleotide sequence accession numbers.

This whole-genome shotgun project has been deposited at DDBJ/EMBL/GenBank under the accession no. JRKX00000000. The version described in this paper is the first version, accession no. JRKX01000000.
